# Porcine synapsin 1: *SYN1* gene analysis and functional characterization of the promoter^☆^^☆☆^

**DOI:** 10.1016/j.fob.2013.10.002

**Published:** 2013-10-07

**Authors:** Claus Hedegaard, Kasper Kjaer-Sorensen, Lone Bruhn Madsen, Carina Henriksen, Jamal Momeni, Christian Bendixen, Claus Oxvig, Knud Larsen

**Affiliations:** a Department of Molecular Biology and Genetics, Aarhus University, Blichers Alle 20, Tjele DK-8830, Denmark; b Department of Molecular Biology and Genetics, Aarhus University, Gustav Wieds Vej 10, Aarhus C DK-8000, Denmark

**Keywords:** GFP, Neuron-specific promoter, Pig, Transgenic, Zebrafish, Ab, antibody, BSG, basal ganglia, BST, brain stem, CBE, cerebellum, Chr, chromosome, CMV, cytomegalovirus, FB, forebrain, FCO, frontal cortex, GFP, green fluorescent protein, HB, hindbrain, HIP, hippocampus, LLG, lateral line ganglion, MB, midbrain, NRSE, neuron restrictive silencer element, OC, optic chiasm, ON, olfactory neuron, R, retina, REST, RE1-silencing transcription factor, TG, trigeminal ganglion, TSS, transcription start site, WPRE, Woodchuck hepatitits virus Post-transcriptional Regulatory Element

## Abstract

Synapsin 1 (SYN1) is a phosphoprotein involved in nerve signal transmission. The porcine *SYN1* promoter orthologue was cloned and characterized to provide a means of expressing a transgene specifically in neurons. The nucleotide sequence of the promoter displayed a high degree of conservation of elements responsible for neuron-specific expression. Expression analysis of *SYN1* demonstrated presence of transcript during embryonic development. Analysis of GFP expression in transgenic zebrafish embryos suggests that the pig *SYN1* promoter directs expression in neuronal cells. Thus, the *SYN1* promoter is a good candidate for use in the generation of pig models of human neurodegenerative disorders.

## Highlights

• The porcine synapsin1 (SYN1) promoter was cloned and characterized.
• SYN1 mRNA expression is detected in brain during embryo development.
• The *SYN1* gene is mapped to pig chromosome X.
• Porcine SYN1 directs GFP expression in neuronal cells of transgenic zebrafish.

## 1 Introduction

Synapsin 1 (SYN1) belongs to a family of phosphoproteins, also comprising synapsin 2 and 3 with isoforms. These synapsins associate with the surface of synaptic vesicles [1,2]. Members of the synapsin family have common protein domains and are implicated in neuronal development, synaptogenesis, and maintenance of mature synapses and modulation of neurotransmitter release [3]. Synapsins regulate synaptic vesicle traffic and are also involved in the regulation of synaptic vesicle availability for release and in short-term plasticity. Two different carboxy-terminal forms of SYN1, a and b, exist, originating from alternative splicing of a common transcript. Well-known from a number of vertebrate species, SYN1 is an important player in neurotransmitter release, axonogenesis and synaptogenesis illustrated by knockout mice presenting with an epileptic phenotype [4,5]. Binding to small synaptic vesicles found in the nerve terminals, SYN1 possibly has an exocytotic regulatory role in linking the vesicles to the cytoskeleton and each other [6–8]. Furthermore, SYN1 is likely involved in neuronal development and formation of synaptic contacts between neurons [9–11]. Mutations in the *SYN1* gene have been demonstrated to be associated with epilepsy [12] and autism spectrum disorders with or without epilepsy [13]. The mutations create changes in the SYN1 protein thereby potentially causing defects in synaptic vesicle traffic and nerve terminal function. In accordance with its native function, SYN1 is found to be brain- and neuron-specifically expressed mediated by the promoter region of the *SYN1* gene [14]. The SYN1 protein serves as a substrate for several different protein kinases and phosphorylation is very likely functioning in the regulation of this protein in the nerve terminal.

In transgenesis, direction and limitation of gene expression to neurons have been demonstrated with the *SYN1* promoter [15–20]. Though being highly specific, the *SYN1* promoter appears relatively weak which has prompted a down-stream addition of a Woodchuck hepatitis virus Post-transcriptional Regulatory Element (WPRE). The resulting transgene expression cassette exhibits retained neuronal specificity and a considerably elevated level of expression of two-threefold [21,22]. High levels of transgene expression are often strived for e.g. in generation of animal models for human diseases to rapidly introduce a disease phenotype. Such expression levels can in general be obtained with viral promoters as that of the widespread human cytomegalovirus (CMV) but constitutive expression, which is undesirable in some studies, and frequent gene silencing are other characteristics of the CMV promoter [23–26]. The utilization of an endogenous promoter would likely overcome the challenge of exogene silencing [27–29] and, hence, secure stable long-term transgene expression [23] presumably required for inducing phenotypes of e.g. age-related, neurodegenerative disorders. Several human diseases of this kind have been modelled in rodents, particularly mice, but improvements are sought for using primates [30–32] and, more recently, pigs [33], as use of swine generally does not face the substantial public concerns related to the use of primates as laboratory animals. In addition, characterization of the porcine genome provides evidence of a close genetic relationship between humans and pigs which together with the well-known anatomic and physiological similarities points to the pig as a good species for modelling human diseases not least those of the central nervous system.

Altogether this has prompted us to clone the porcine *SYN1* promoter and characterize the expression pattern and cell specificity expected from transgene constructs including this promoter.

## 2 Materials and methods

### 2.1 Ethic statements

The pigs were housed and used in compliance with European Community animal care guidelines. Beforehand, the experimental procedures were approved by the National Ethical Committee in Denmark (Approval No. 2010/561-1891). Pigs were sacrificed by an injection with 30 mg/kg Pentobarbital (Vipidan, Denmark). Experiments involving zebrafish were carried out in accordance with the recommendations from the European network on fish biomedical models and according to Danish legislation. All zebrafish used in this study were under the age of 72 h and hence the experiments do not require any approval. Zebrafish embryos were killed by a tricaine overdose.

### 2.2 Biological subjects

The study included pig embryos sampled at 60, 80, 100, and 115 days of gestation. Five different brain areas were included in this study: cerebellum (CBE), frontal cortex (FCO), brain stem (BST), basal ganglia (BSG) and hippocampus (HIP). Three separate tissues were applied for each type of brain tissue and time in gestation, yielding a total of 60 samples. Brain tissue samples were also collected from three Danish Landrace pigs, aged 1–2 years and weighing 125–200 kg.

### 2.3 Cloning of the *SYN1* promoter

The 5′-flanking sequence of the human *SYN1* gene (GenBank: M55301) was compared to preliminary porcine genomic data which identified a homologous sequence from which a forward primer (PromFW 5′-AAAGGGATGGGGGCGTAC-3′) was designed. The corresponding reverse primer (PromRV 5′-ATGAAGTTGCTGTCCGACAG-3′) was derived from the putative exon 1 of a porcine EST sequence displaying homology to the human *SYN1a* encoding sequence (GenBank: NM_006950) (DNA Technology, Aarhus, Denmark). For amplification of the promoter, porcine genomic DNA purified from boar semen was used as template in a PCR with a Long PCR Enzyme Mix applied together with the standard buffer (Fermentas, Glen Burnie, MD) and the temperature conditions: 94 °C for 30 s; 35 cycles of 94 °C for 10 s, 64 °C for 30 s, and 68 °C for 1.30 min; 68 °C for 10 min. The amplicon was cloned in electrocompetent One-Shot *Escherichia coli* with the TA TOPO Cloning Kit from (Invitrogen, Carlsbad, CA). Complete Sanger sequencing of the insert in both directions using a Big Dye Terminator Cycle Sequencing Kit and an ABI 3730 xl DNA Analyzer (Applied Biosystems, Warrington, UK) was carried out by primer walking.

### 2.4 Expression analysis of *SYN1*

**Fig. 1:**
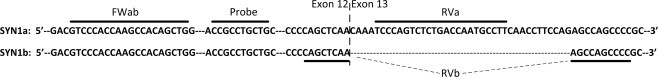
Parts of exon 12 and 13 in the 3′ end of porcine *SYN1a* and *SYN1b* coding sequences. For expression analysis of the two separate mRNAs, a qPCR assay was designed. The forward primer (FWab) and probe were common to both messengers whereas specific reverse primers (RVa and RVb) were created for discrimination of the mRNAs.

Splice variants of *SYN1* (a and b) described in other species have also been found in the pig [34] and enabled the design of PCR primers discriminating between the two isoforms (Fig. 1). A *SYN1a*-specific reverse primer (CdsRVa 5′-AGGCATTGGTCAGAGACTGGG-3′) was placed in the 5′ terminal, unique part of exon 13 whereas a syn Ib-specific reverse primer (CdsRVb 5′-GGGGCTGGCTTTGAGCTG-3′) spanned the junction between exon 12 and the truncated exon 13. A generic forward primer in exon 12 (CdsFWab 5′-GTCCCACCAAGCCACAGCT-3′) was used with both of the reverse primers (DNA Technology, Aarhus, Denmark) and a common 5′-GCCTGCTG-3′ LAN probe (#40, Human Probe Library, Exiqon) detected both SYN 1a and 1b amplicons. *GAPDH* served as reference gene for normalization of data due to its appropriateness found in an evaluation with other candidate genes [35]. Primers (GAPDHFW 5′-GACTCATGACCACGGTCCATG-3′, GAPDHRV 5′-GTCAGATCCACAACCGACACG-3′) amplified a fragment of the *GAPDH* coding sequence detected with the probe 5′-VIC-CATCACTGCCACCCAGA-3′.

Total RNA was purified from frontal cortex (FCO), cerebellum (CBE), brain stem (BST), hippocampus (HIP), and basal ganglia (BSG) from pig foetuses recovered 60, 80, 100, and 115 days post artificial insemination, respectively. cDNA was synthesized with the reverse transcriptase Superscript III primed by random hexameric nucleotides (Invitrogen, Carlsbad, CA). The cDNA libraries were used as templates in real-time PCRs quantifying *SYN1a*, *SYN1b*, and *GAPDH* transcripts, respectively, in a TaqMan based assay. The cDNA preparation from frontal cortex at 115 days was diluted 2, 4, 8, 16, and 32 times to produce a series for semi-quantitative calculations.

The equality of *SYN1a* and *SYN1b* expression levels between different times of gestation within the five sampled tissues was tested for statistical significance using the standalone software REST [36]. The statistical model applied was the Pair Wise Fixed Real location Randomization Test. The assumption regarding normal distribution of the data was avoided and differences in expression between groups were assessed using the means for statistical significance by randomization. The level of probability was set at *P* < 0.05 as statistical significance and 50,000 randomization steps were implemented in each comparison.

### 2.5 Engineering of *SYN1* DNA constructs for zebrafish transformation

The Tol2-SYN1promoter:GFP plasmid was constructed based on the pT2AL200R150G vector kindly provided by Koichi Kawakami, National Institute of Genetics, Japan [37,38]. The restriction enzymes *Xho*I and *Hin*dIII (New England BioLabs) were used to replace the EF1a-promoter, originally placed in the pT2AL200R150G vector, with the porcine *SYN1* promoter. Using PCR, linkers for subsequent cloning were added to the porcine *SYN1* promoter sequence (GQ168794). PCR was carried out with the primers: pSYN1-*Xho*I: 5′-CCGCTCGAGCGGAGACCAAATGTGTGTGTGTAG-3′ and pSYN1-*Hin*dIII: 5′-CCCAAGCTTGGGGCGGCGCCGCAGGTAGTTCATG-3′. The amplified product was cloned into a TOPO vector (Invitrogen) and digested with the restriction enzymes *Xho*I and *Hin*dIII. The *SYN1* insert was cloned into a *Xho*I and *Hin*dIII digested pT2AL200R150G vector.

In order to substitute the nucleotide A with a C in position −230, i.e. 230 nucleotides upstream of the transcription start site, of the porcine *SYN1* promoter sequence (GQ168794), an equivalent to position site-directed mutagenesis was performed employing the QuickChange® XL Site-Directed Mutagenesis Kit (Stratagene). PCR was accomplished in accordance with the manufacturer's recommendation applying the following primers: SYNP-MUTF: 5′-GCGCACTGTCGTCTTCCGCACCGCGGACAGCGC-3′ and SYNP-MUTR: 5′-GCGCTGTCCGCGGTGCGGAAGACGACAGTGCGC-3′ and the pT2AL200R150G vector harbouring the *SYN1* promoter. The PCR conditions were: Denaturation at 95 °C for 1 min, 18 cycles of 95 °C for 50 s, 60 °C for 50 s and 68 °C for 2 min. The PCR program was concluded by an extension at 68 °C for 7 min. To ensure that the mutation of interest was integrated in the porcine *SYN1* promoter, several colonies were picked and grown overnight and plasmids were harvested and sequenced according to standard procedures. Plasmid DNA for microinjection was purified from a culture of transformed DH5a cells (Invitrogen) using the QIAprep Spin Miniprep Kit (Qiagen).

The resulting *SYN1* constructs, Tg(pSYN1:GFP) = SYN1pwt (−230A) and Tg(pSYN1Mut:GFP = SYN1pMut (−230C) were used for transformation of zebrafish.

### 2.6 Handling of zebrafish

Zebrafish of the AB strain were obtained from the Tübingen zebrafish stockcenter. The fish were fed twice a day and kept at 28.5 °C on a 14 h light/10 h dark cycle. The embryos were obtained by natural crosses, reared in E3 buffer (5 mM NaCl, 0.17 mM KCl, 0.33 mM MgSO_4_, 10^−5^% methylene blue, 2 mM Hepes pH 7.0), and staged according to Kimmel et al. [39]. Upon completion of gastrulation, the E3 buffer was supplemented with 0.003% *N*-phenylthiourea (PTU) (SIGMA) to inhibit pigmentation.

### 2.7 Preparation of Tol2 transposase mRNA

The pCS-zT2TP plasmid [37,38] kindly provided by Koichi Kawakami, National Institute of Genetics, Japan, was linearized with *NotI* and used as a template for *in vitro* transcription of the Tol2 transposase mRNA. Capped mRNA for microinjection was synthesized using the mMessage mMachine SP6 Kit (Ambion, Inc.). The RNA synthesis reaction was treated with TURBO DNase (Ambion) followed by purification using the RNeasy MinElute Cleanup Kit (Qiagen). RNA quality was assessed by denaturing RNA gel electrophoresis using the FlashGel System (Lonza), and RNA content was quantified by spectroscopy.

### 2.8 Micro-injection of zebrafish and GFP expression

Micro-injection volumes were measured and calibrated by performing 10 injections into a 0.5 μL microcapillary tube (Drummond Microcaps), measuring the amount of liquid using a ruler, and calculating the volume per injection. Five nanoliters of injection mixture containing 50 pg Tol2 transposase encoding mRNA and 100 pg Tol2-SYN1promoter:GFP plasmid were microinjected into the centre of the yolk of zebrafish zygotes.

For microscopy, live embryos were sedated with 150 ng/mL tricaine (Aldrich) in E3/PTU and mounted in 1.5% hydroxypropyl methyl cellulose M_n_ 86000 (Sigma–Aldrich). GFP expression in zebrafish live embryos and larvae was documented using a Zeiss AXIO Observer.D1 microscope equipped with Zeiss Colibri Illumination System and Zeiss AxioCam MRm. Fluorescence microscopy of immunostained embryos was documented using a Zeiss AXIO Observer.Z1 equipped with Zeiss Colibri .2 Illumination System, Zeiss Apotome .2, and Zeiss AxioCam HRm. Images were stacked and Z-projections were made in ImageJ. Contrast and brightness of Z-projections were adjusted and the resulting images were merged in Adobe Photoshop CS5.

### 2.9 Whole mount immunohistochemistry

Zebrafish embryos were euthanized, fixed and immunostained as described previously (REF: PMID 23430244). Primary antibodies: Ab1-tuba (6-11B-1, Sigma–Aldrich) (1:2000) and anti-GFP, rabbit polyclonal antibody, unconjugated (Invitrogen) (1:500). Secondary antibodies: Alexa Fluor 555 donkey anti-mouse (Invitrogen) (1:1000) and Alexa Fluor 488 goat anti-rabbit (Invitrogen) (1:1000).

### 2.10 Detection of transgene

The upper half of the tail-fin was cut from potential transgenic and control zebrafish and stored at −20 °C in RNA*later*® reagent (Ambion). Genomic DNA and RNA of tail fin cuts from eleven transgenic zebrafish and two controls were extracted using TriReagent (SIGMA).

Synthesis of cDNA was conducted with 750 μg of total RNA isolated from the tail fin using SuperScript III RNase H^−^ reverse transcriptase (Invitrogen) and random hexamer primers according to the manufacturer's recommendations.

Detection of the transgene was performed by PCR amplification of fragments covering the *GFP* gene and the porcine synapsin promoter sequence. PCR reactions were done according to standard protocols in a volume of 10 μl with ng DNA, 1 μM of each primer, 0.5 mM dNTP and x0.2 U Phusion DNA polymerase (Finnzymes).

Primers used to amplify the GFP fragment (720-bp) were GFP-PS-F: 5′-ATGGTGAGCAAGGGCGAGG-3′ and GFP-PS-R: 5′-TTACTTGTACAGCTCGTCCATGCC-3′.

Primers used to amplify the SYN1 promoter sequence (576 bp) were: SYNPp1f: 5′-CGTGAGTGTAGGCAGGCATGCCCAT-3′ and SYNPp1r: 5′- ATGCGCAATTTGGGGAGTGGGGGCGG -3′. The PCR conditions were: Denaturation at 98 °C for 30 s, 10 cycles of touchdown (−0.5 °C/cycle) 98 °C for 30 s, 65–60 °C for 30 s and 72 °C for 1 min 35 s, 25 cycles 98 °C for 30 s, 60 °C for 30 s, 72 °C for 1 min 35 s, The PCR program was concluded by a 7 min extension at 72 °C.

The RT-PCR reaction mix contained: 0.1 μL cDNA, 1.5 mM MgCl_2_, 0.5 mM dNTP, 0.5 μM of each primer GFP-PS-F: 5′-ATGGTGAGCAAGGGCGAGG-3′ and GFP-PS-R: 5′-TTACTTGTACAGCTCGTCCATGCC-3′ and 1 U Phusion DNA polymerase (Finnzymes) in a total volume of 10 μL. The PCR profile was as follows: 98 °C for 30 s, 10 touchdown cycles of 98 °C for 30 s, 68 °C for 30 s, 72 °C for 1 min 35 s, followed by 25 cycles of 98 °C for 30 s, 63 °C for 30 s, 72 °C for 1 min 35 s and finally an elongation at 72 °C for 7 min.

### 2.11 Methylation status of SYN1

In brief, the methylation status of SYN1 was performed by library preparation, sequencing, mapping and analysis. DNA from each sample was extracted and sheared to a size of 200–300 bp using the Covaris Adaptive Focused Acoustics™ (AFA) process (Covaris). Double-stranded DNA fragments were end repaired, A-tailed, and ligated to methylated Illumina adaptors. Ligated fragments were bisulfite converted using the EZ-DNA Methylation-Kit (Zymo research). Following PCR enrichment, fragments of 325–425 bp were size selected and sequenced using Hiseq 2000 Illumina sequencing system.

We used Novoalign short read aligner (version 2.07.12 http://www.novocraft.com/) to align reads to a reference genome. Novomethyl (Beta.8.0 http://novocraft.com/main/page.php?s=novomethyl) was used to call the consensus sequence, identify cytosines and call their methylation state or percentage of cytosines methylated. For finding the methylation percentage of special genes or sequences from our methylome data file, we used Tabix [40].

### 2.12 Analysis of DNA methylation in pig brain at two developmental stages

DNA was isolated from sections of cerebellum and frontal cortex collected from pig brain at 60 days of gestation and from an 11-year-old pig, both Danish Landrace. Genomic DNA from snap-frozen brain samples was extracted using a standard protocol. One microgram of DNA was bisulfite modified using the EZ DNA Methylation Kit (ZYMO Research Group). Modified DNA was purified using the EZ Bisulfite DNA Clean-up Kit (ZYMO Research Group).

Bisulfite sequencing was carried out on a 104 bp *SYN1* sequence. Bisulfite modified DNA was PCR amplified using primers designed with MethPrimer (http://www.urogene.org/methprimer/index1.html) SYNP1P-U2F: 5′-TGGTTTAGTTGGATTGTATTATATGG-3′ and SYNP1P-U2R: 5′-CTCCCGCTACAAACTAAAACAA-3′, PCR was carried in a total volume of 10 ml containing10 ng bisulfite treated DNA, 5 pmol of each primer, 0.5 mM dNTP and 0.625 U PfuTurbo C_x_ HotStart DNA polymerase (Agilent Technologies).

PCR conditions were: 95 °C for 2 min, 30 cycles of 95 °C for 30 s, 52 °C for 30 s, 72 °C for 1 min, and an elongation step of 72 °C for 10 min.

PCR products (104 bp) were gel-purified and cloned using the TOPO TA Cloning Kit for Sequencing (Invitrogen, Denmark). For each tissue 10 clones were randomly selected and plasmid DNA was prepared. DNA was sequenced in both directions using the vector-specific primers TOPO-F: 5′-AAGGGGGATGTGCTGC-3′ and TOPO-R: 5′-GCTCACTCATTAGGCAC-3′ using the BigDye terminator cycle sequencing kit and a 3730 Genetic Analyzer (Applied Biosystems).

## 3 Results

### 3.1 Characterization of porcine SYN1 genomic sequence

A blast search in the Pig Genome v.10 sequence database (http://www.animalgenome.org/blast/blast.php?bdb=pig10) using the porcine *SYN1* cDNA sequence (GenBank: NM_001141988) revealed a 53 kb sequence covering the entire *SYN1* gene (GenBank: JN673714). The intron–exon structure is presented in Table S1. The porcine *SYN1* gene is composed of 13 exons with very non-uniform sizes ranging from 58 to 610 nucleotides (Table S1). All the observed splice acceptor and donor sites were in accordance with the consensus GT–AG rule (Table S1). The genomic organization of *SYN1* genes seems to be well conserved. All exons in the porcine *SYN1* gene, except for exon 12, have the same length of coding sequence as those of the human *SYN1* sequence [41]. The additional 21 nucleotides found in the porcine sequence (exon 12) have been documented earlier [34]. In addition, the lengths of most introns of the porcine *SYN1* gene were comparable to the human counterparts. Similar to human *SYN1*, the porcine *SYN1* exon13 also contains two splice acceptor sites [1]. The two different AG splice acceptor sequences are localized at positions 50753 and 50793, respectively, in the deposited *SYN1* sequence (GenBank: JN673714) and give rise to messages for *SYN1a* and *SYN1b* as shown in Fig. 1. This particular splice mechanism of *SYN1* is conserved between the pig, human, bovine and rat mRNAs.

### 3.2 Sequence analysis of the *SYN1* promoter

**Fig. 2:**
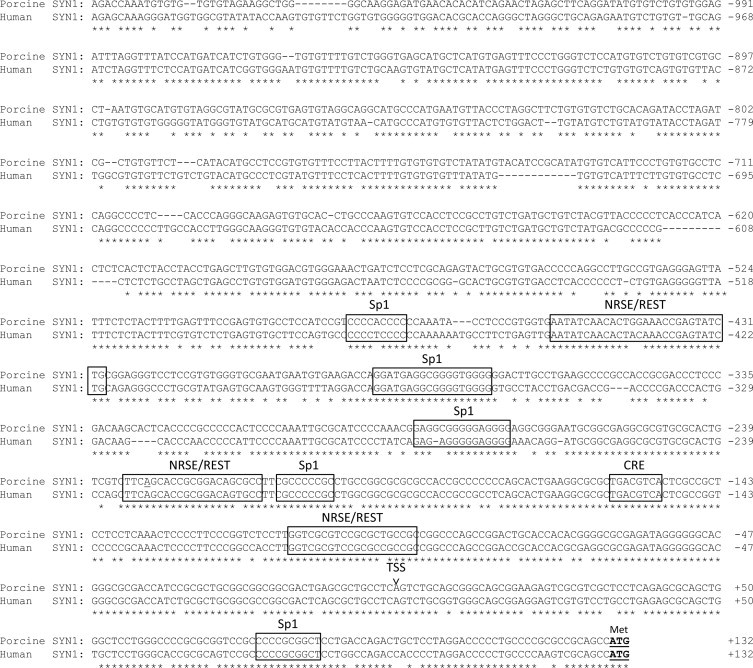
Alignment of the porcine and human (GenBank: M55301) *SYN1* promoters using ClustalX2. Extensive homology is observed in particular in the 3′ end with the start codon (marked with a box and an ‘M’), around the putative transcription start site (indicated by an arrow), and within the regulatory elements NRSE/REST, Sp1, and CRE (marked with boxes). The nucleotide A at position −230 mutagenized to a C in the experiments with transgenic zebrafish is underlined.

We have PCR amplified, cloned and sequenced a 1226 bp fragment of the 5′ flanking region/putative promoter of the porcine *SYN1* gene (GenBank: GQ168794). Aligning of the obtained nucleotide sequence, trimmed at the 3′ end to a total of 1208 bp, with the human *SYN1* promoter (Fig. 2) revealed an overall identity of 79%. The degree of homology decreased with increasing distance in the 5′ direction from the start codon illustrated by a 91% identity of the 3′ terminal 366 bp (−233 bp from the putative transcription start site (TSS)). The TSS of porcine *SYN1* was predicted by comparison with the human *SYN1* mRNA sequence (GenBank Access. No. M55301). Hence, the nucleotide in position +1 is an A which is common in vertebrates. In addition to the nucleotide substitutions, a number of indels are present exclusively within the 5′ terminal less conserved segment of 842 bp.

The nucleotide sequence of the genomic DNA 1226 bp upstream of the transcription start site (TSS) of the porcine *SYN1* gene was analyzed for transcription factor binding sites using the computer-based MatInspector and TFSEARCH program (http://molsun1.cbrc.aist.go.jp/htbin/nph-tfsearch) and using the transfac database. The analysis revealed neither a TATA box nor any CCAAT box in the 1208 bp 5′-flanking sequence of porcine *SYN1*. Instead, the *SYN1* promoter was found to be of a GC-rich type with a pronounced GC-content of 76% within the 3′ terminal 366 bp (−233 bp from the putative TSS) as compared to the overall GC-content of 62%. Furthermore, the distance from the putative TSS to the translation initiation codon (position 1232–1234 in Fig. 2) was 130 bp accounting for the 5′ untranslated region of the *SYN1* mRNA. The putative TSS and the transcription factor binding elements, Sp1 (of which five are present) and CRE (cAMP responsive element), identified in the human promoter are found to be completely conserved in the porcine promoter. Moreover, a fourth element of 21 bp, NRSE (neuron restrictive silencer element) also named the RE1-silencing transcription factor/neuron-restrictive silencer factor (REST/NRSF), involved in limiting gene expression to neurons, was found in three positions in the porcine *SYN1* promoter. The sequences of the RE1 elements within the *SYN1* promoter are highly conserved between pig and human.

In conclusion, a high degree of sequence homology between the porcine and human *SYN1* promoters was demonstrated. Importantly, transcription factor binding elements, the sequences surrounding the TSS, and the absence of TATA- and CAAT boxes were conserved in the porcine promoter. The high sequence similarity between human and porcine *SYN1* could indicate the existence of similar mechanisms for regulation of expression.

### 3.3 The *SYN1* gene localises to chromosome X

Recently, we have used Blat software to localize the *SYN1* gene in the Sus scrofa 10.2 genome [42]. The *SYN1* gene maps to SsChrX: 47,336,723–47,388,638 (Table 1). The human and mouse *SYN1* genes have been mapped to the X chromosomes of these species [43,44].

### 3.4 *SYN1* is abundantly expressed prenatally

The expression level and pattern of *SYN1* mRNA were investigated in various porcine brain tissues (FCO, CBE, BST, HIP, and BSG), at various prenatal times (60, 80, 100, and 115 days of gestation), and in technical and biological triplicates in a semi-quantitative PCR assay relative to *GAPDH* expression. The specificity of the separate reverse primers discriminating between the isoforms *a* and *b* of the *SYN1* transcript was validated by electrophoretic determination of amplicon length difference prior t the expression analysis (data not shown).

**Fig. 3:**
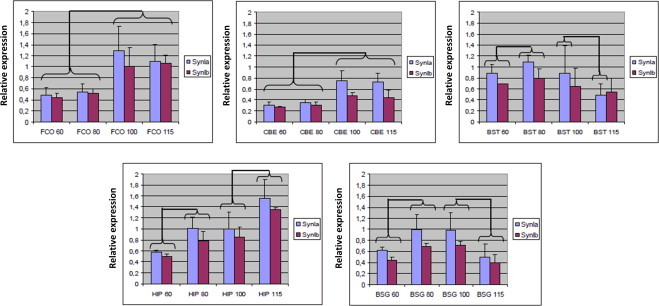
Analysis of endogenous porcine *SYN1a* and *SYN1b* expression levels by qPCR normalized to *GAPDH* expression. Five tissues, frontal cortex (FCO), cerebellum (CBE), brain stem (BST), hippocampus (HIP), and basal ganglia (BSG), at four different prenatal times (embryonic day 60, 80, 100, and 115), and in biological triplicates were included. Statistically significant different expression levels were indicated by connective lines.

Both *SYN1a* and *SYN1b* are expressed in all five brain tissues at all developmental stages examined even as early as 60 days of gestation (Fig. 3). However, for both *SYN1a* and *SYN1b* transcripts there are a considerable degree of heterogeneity among animals which is reflected by the standard deviations. Compared to the constitutive *GAPDH* expression, the levels of *SYN1a* ranged from 0.32 to 1.56 and of *SYN1b* from 0.27 to 1.35. Also, the *SYN1* expression developed differently over time in the investigated tissues.

In FCO and CBE, the *SYN1* mRNA expression levels were significantly higher at day 100 and 115 of gestation compared to day 60 and 80 (*SYN1*a: FCO, *P* ≤ 0.001 and CBE, *P* ≤ 0.006; *SYN1b*: FCO, *P* ≤ 0.001 and CBE *P* ≤ 0.009) yielding an increase of 1.5–2 fold in level of expression. Moreover, there is no differential expression of any of the variants neither in FCO nor in CBE between day 60 and 80 of gestation (*SYN1a*: FCO, *P* = 0.39 and CBE, *P* = 0.24; *SYN1b*: FCO, *P* = 0.06 and CBE, *P* = 0.46) and day 100 and 115 of gestation (*SYN1a*: FCO, *P* = 0.32 and CBE, *P* = 0.60; SYN1b: FCO, *P* = 0.68 and CBE, *P* = 0.53). The tendency of an increase in expression level of the *SYN1* messenger variants over time is also present in HIP. Here the significant up-regulation is present between day 60 and 80 of gestation and between day 100 and 115 of gestation (*SYN1a*: *P* ≤ 0.001 and *P* ≤ 0.001, respectively; *SYN1b*: *P* ≤ 0.001 and *P* < 0.000, respectively). For BST and BSG, the level of *SYN1* expression, as in HIP, also increases between day 60 and 80 of gestation (*SYN1a*: BST, *P* = 0.038 and BSG, *P* = 0.008; *SYN1b*: BST, *P* = 0.036 and BSG, *P* = 0.003). However, in both tissues, except for BST *SYN1b*, there is a significant decrease in the level of expression between day 100 and 115 of gestation (*SYN1a*: BST, *P* = 0.037 and BSG, *P* = 0.002; *SYN1b*: BSG, *P* = 0.002). In summary, expression of porcine *SYN1* mRNA was found in all investigated prenatal brain tissues and in considerable amounts reaching ∼1.5 times that of GAPDH but also found to diverge substantially in time and between different tissues. In general, the expression levels of *SYN1a* and *SYN1b* were comparable, albeit the level of *SYN1b* tends to be slightly lower than that of *SYN1a.*

### 3.5 *In vivo* assessment of *SYN1* promoter activity

**Fig. 4:**
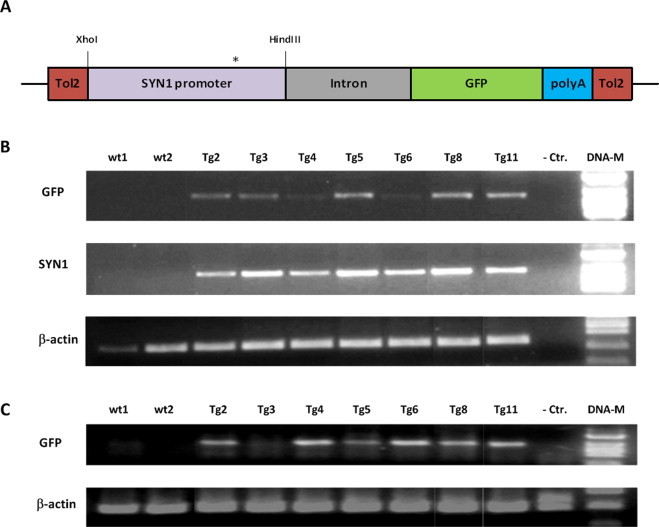
Detection of the *SYN1* transgene in genomic DNA purified from tail cuts of two non-transgenic zebrafish (lanes 1 and 2 from left) and seven transgenic zebrafish (lanes 3–9). (A) DNA construct used for transgenesis of zebrafish. A modified construct with an A to C substitution (nucleotide position 847) in the NRSE element is shown by the asterisk. (B) Examination for presence of transgenes in injected zebrafish. PCR reactions were performed using specific primer sets for the GFP gene sequence (upper panel) and the SYN1 promoter (middle panel). A *β-actin* specific primer set establishing the quality of the genomic DNA is shown in the lower panel. All of the potentially transgenic zebrafish proved to contain the transgene being positive for both GFP (720-bp band) and SYN1 (576-bp band). A negative reagent control is shown in lane 10 and a DNA marker is seen in lane 11. As expected no transgene was detected in the non-transgenic zebrafish. (C) GFP transcript analysis of transgenic zebrafish by RT-PCR. All transgenic zebrafish appear to express GFP transcript, although at different levels, whereas no expression is seen in wild-type fish.

To assess promoter activity and specificity *in vivo*, we injected zebrafish embryos with a plasmid expressing GFP driven by a 1.2 kb fragment from the porcine *SYN1* promoter. We made use of the *Tol2* transposon system (ref: PMID:19504063) enabling efficient stable genomic integration allowing for increased rate of integration and reduced non-specific expression compared to injection of a standard expression plasmid [37,38]. The DNA construct used for fish transformation is based on the pT2AL200R150G vector (kindly provided by Koichi Kawakami, National Institute of Genetics, Japan) (ref: PMID: 16959904, PMID: 15239961). The final construct, pT2AL:SYN1:GFP, used for microinjection into fertilized zebrafish eggs, is shown in Fig. 4A.

GFP positive injected embryos were raised to adulthood and finclips were tested for the presence of the transgene by PCR analysis using primer sets amplifying a sequence of the *SYN1* promoter and the GFP coding sequence, respectively. As shown in Fig. 4B, finclips from seven out of eleven adults with observed GFP expression in the -ic stage contained the transgene. Very faint signals were obtained in transgenic zebrafish Tg4 and Tg6 with PCR amplifying the GFP transgene. Sequencing of the amplified DNA fragments confirmed the identity of *SYN1* and *GFP*. The copy number of the transgene *SYN1* in the transgenic zebrafish was established by qPCR and found to be in the range 1–100 copies between individuals (data not shown). RT-PCR analyses showed that *GFP* transcript was detected in most of the transgenic fish although at a much lower level as compared to the level of *β-actin* (Fig. 4C). The low expression in certain transgenic zebrafish could eventually be explained by gene silencing. Sequencing of the RT-PCR product confirmed the identity of GFP. Control reactions with RNA as a template in the PCR did not amplify any products (data not shown).

**Fig. 5:**
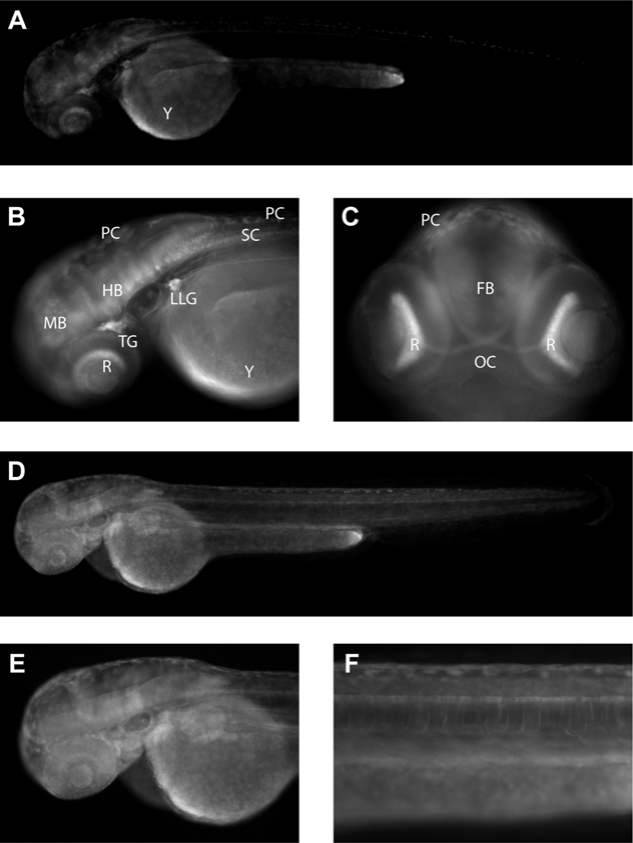
F1 transgenic zebrafish embryos expressing GFP under the regulation of the wild-type and NRSE-mutated porcine SYN1 promoters. Embryos oriented anterior to the left, dorsal to the top (A,B,D,E,F) or in an anterior view, dorsal to the top (C). All pictures are representative of GFP positive Tg(pSYN1:GFP) embryos (*n* > 50) at 72 hpf (A–C) or Tg(pSYN1-MUT:GFP) embryos (*n* = 8) at 48 hpf (D–F). Non-specific autofluorescence is observed from the yolk (Y) and pigment cells (PC). (A) Whole embryo view showing distinct but weak GFP expression in neuronal tissues including brain and spinal cord (SC). No expression is observed in non-neuronal tissues. (B) Close-up view showing GFP expression in midbrain (MB), hindbrain (HB), spinal cord, retina (R), trigeminal ganglion (TG), and posterior lateral line ganglion (LLG). Note the segmented signal in the hindbrain highlighting neurons of the rhombomeres, and the neuronal cell bodies discernible in the trigeminal and lateral line ganglia. Also, the bundle of axons projecting caudally from the lateral line ganglion are visible dorsal to the yolk sac. (C) Anterior view showing GFP expression in retina and optic chiasm (OC) and more weakly in the forebrain (FB). (D–F) The mutated promoter drives broad, possibly ubiquitous expression in zebrafish embryos.

To evaluate germ line transmission and GFP expression pattern in fully transgenic embryos, adults from the injected generation were crossed to wild type. In addition, we examined the expression pattern of wild type and mutant (A-230C mutation in the NRSE element) *SYN1* promoter. Embryos with weak neuronal GFP signals were found in the F1 generation descending from five pSYN1-injected individuals (Fig. 5A–C), and embryos with broad, possibly ubiquitous expression was identified from a single founder injected with the NRSE-mutated promoter construct (Fig. 5D–F). Expressing tissues in Tg(SYN1:GFP) embryos include forebrain, midbrain, hindbrain, spinal cord, retina, optic chiasm, trigeminal ganglion, posterior lateral line ganglion, and olfactory neurons (Fig. 5B, C, and Supplementary Fig. 1). Note the segmented signal in the hindbrain highlighting neurons of the rhombomeres, the neuronal cell bodies discernible in the trigeminal and lateral line ganglia, and the bundle of axons projecting caudally from the lateral line ganglion (Fig. 5B). No expression was observed in non-neuronal tissues. At 26 hpf, co-immunostaining for GFP and acetylated tubulin revealed that the promoter-driven GFP expression co-localized with the neuronal marker in embryos injected with the wild-type pSYN1:GFP construct (Supplementary Fig. 2). Our in vivo analysis of the NRSE mutated *SYN1* promoter underlines the functional requirement for this element, strongly suggesting that residue A230 in the NRSE element is required for suppression of non-neuronal expression.

### 3.6 Methylation status of the *SYN1* gene

The methylation status of the porcine *SYN1* gene was examined by whole-genome bisulfite sequencing. Approximately 52 kb of the coding sequence of the *SYN1* gene was investigated for two different porcine tissues: occipital cortex and liver. In occipital cortex 5232 methylated CpG reads were detected out of a total of 7948 reads yielding a methylation degree of 66% (Table 1). Similarly, in liver tissue approx. 14,000 methylated reads were seen in a total of 25,500 reads, i.e. a methylation degree of 55%. In conclusion, the methylation degree is significantly higher in brain tissue compared with liver, using the chi-square test (*p*-value < 0.001). A 104 bp DNA stretch in the *SYN1* promoter was also examined for methylation. Only two reads out of 275 were found to be methylated in brain tissue yielding a methylation degree of 0.7%, i.e. very close to zero. Similarly, only two reads out of 1453 reads were identified in liver tissue.

**Fig. 6:**
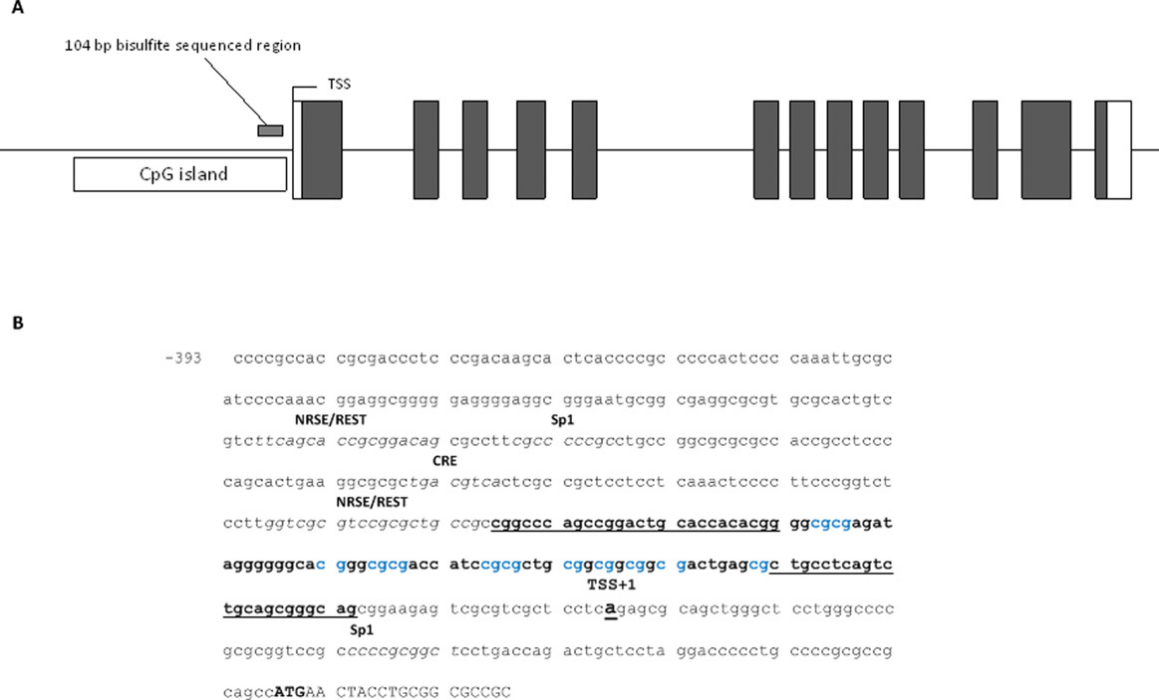
Bisulfite sequencing of a selected region of the porcine *SYN1* promoter. (A) Schematic representation (not drawn to scale) of the porcine *SYN1* gene exon–intron structure with CpG island location. Also, the bisulfite sequenced region of the promoter is indicated. (B) Sequence of the *SYN1* promoter region selected for bisulfite sequencing. The analyzed sequence is shown in bold letters. Primer sequences are underlined. Twelve CpG dinucleotides are marked in blue letters. The transcription start site (+1) and the ATG start codon (bold capitalized letters) are also indicated. (For interpretation of the references to colour in this figure legend, the reader is referred to the web version of this article.)

To investigate whether the promoter of the porcine *SYN1* promoter is enriched for CpGs, and to determine its methylation status, the overlap between these features was determined. The location of CpGs in the promoter was identified using a sliding window operation applying the Takai–Jones CpG criteria [45]. The Takai–Jones criteria were as follows: length > 500 bp, GC > 55%, CpG observed/expected ratio > 65%. In silico analysis of the porcine *SYN1* promoter sequence using MethPrimer (http://www.urogene.org/methprimer/index1.html) revealed one CpG island of approx. 660 nucleotides upstream of the ATG start codon (Fig. 6A). A discrete region of the *SYN1* promoter sequence covering position −22 to −133 was selected for bisulfite sequencing (Fig. 6B). This region is extremely GC-rich (76%; 44 G, 38C, 16A and 9T) and contains 12 CpG dinucleotides. Bisulfite sequencing was carried out on DNA isolated from cerebellum and frontal cortex samples from pig embryos at 60 days of gestation and from a 12 year old pig. PCR was successfully amplified from bisulfite treated DNA and sequenced. No methylation in the selected DNA stretch of the *SYN1* promoter was observed in the different tissues. Comparison of methylation degree between 60 day old foetuses and a 12 year old sow revealed no methylation neither in FCO nor CBE in the 12 year old pig. Similarly, PCR amplification of bisulfate treated DNA samples from frontal cortex and cerebellum yielded no methylation of CpGs.

## 4 Discussion

The porcine orthologue of the *SYN1* promoter was cloned, sequenced, and characterized with respect to regulatory elements, genomic localization, and expression activity.

The porcine *SYN1* promoter seems analogous to both the human and murine promoters in all relevant aspects i.e. conservation of (i) transcription factor interacting segments, (ii) the regions flanking the putative TSS, (iii) the sequence 429 bp upstream of the putative TSS including GC-overrepresentation. Early studies on SYN1 gene structure and function reported only 225 bp 5′ to the TSS to act as a minimal promoter and facilitate neuron-specificity in cell lines [41,46,47]. Subsequently, a large promoter fragment of ∼4.3 kb was shown *in vivo* to direct transgene expression almost exclusively to neuronal tissues and also to correlate in a developmentally regulated way with endogenous *SYN1* expression [20]. A following comprehensive *in vitro* study confirmed the short 225 bp fragment harbouring the NRSE/REST to be sufficient to obtain neuronal specificity and further strongly indicated the NRSE/REST to be solely responsible for generating the specificity [14]. In addition, it was demonstrated that an even shorter 199 bp fragment without the NRSE held constitutive promoter activity in itself, but also that the segment immediately upstream of the NRSE (422 bp in total) substantially enhanced the derived expression levels. In the present study we demonstrated that a substitution of a single nucleotide, A-230C in the NRSE changed the neuron-specificity of SYN1. Hence, the mutated nucleotide is required for suppression of non-neuronal expression. Recent studies by Paonessa et al. [47] have revealed several *cis*-elements for the transcriptional activator Sp1 of which some are in close proximity to RE-silencing transcription factor (REST) binding motifs. Furthermore, it was demonstrated that REST directly inhibits Sp1-mediated transcription which leads to SYN1 down-regulation [47]. A low level of REST allows a high stability of Sp1 binding to GC boxes eventually leading to increases in *SYN1* transcription. A high level of REST in non-neuronal tissue might therefore explain low levels of *SYN1* expression.

Based on these results, the analogy of the porcine promoter, and the fact that the porcine promoter can be considered an evolutionary intermediate between human and rodents, the porcine *SYN1* promoter could consequently be expected to hold the equivalent ability of limiting gene expression strictly to cell populations of neuronal origin when applied in transgenesis. Furthermore, in experiments utilising systems for gene transfer with limited capacity of transgene construct carriage, e.g. some viral vectors, a fragment of the porcine *SYN1* promoter of only 429 bp, or even shorter (233 bp), could advantageously be applied without compromising neuron-specificity.

Using a cell line hybrid panel and a PCR selective for the *SYN1* promoter, the porcine *SYN1* gene was reliably found to reside on chromosome X most likely in the region p11-13 (data not shown). This is in perfect accordance with the mapping of human and mouse *SYN1* to chromosome X p11.23 and chromosome X A1-4, respectively [44]. More recently, the human and murine genome projects have confirmed the chromosomal localization fitting the previously recognized phenomenon of the X chromosome to be largely conserved between mammalian species in contrast to the autosomal counterparts [47]. In female mammals, one of the X chromosomes is inactivated during early embryonic development by among other mechanisms, DNA methylation of cytosine residues in the 5′ end of genes [48,49]. This holds implications for interpretation of *SYN1* expression levels since the observed transcription levels originate from only one allele as compared to normally two for autosomally localized genes.

By means of semi-quantitative real-time PCR, the expression levels of porcine *SYN 1a* and *1b* were determined in various brain tissues at various prenatal times. Ranging from 0.27- to 1.56-fold of the GAPDH level, the *SYN1* expression levels seem on the one hand to be surprisingly high bearing in mind that the transcription stems from a single allele, that the transcription of *SYN1a* and *1b* has been assessed individually, and that the *SYN1* promoter is considered to be relatively weak [21]. On the other hand, SYN1 is very abundantly present in the central nervous system accounting for at least ∼0.5% of total protein in the brain [50]. In this context, therefore, it should be stressed that transcriptional levels of a gene and the translational levels of the corresponding protein(s) do not necessarily correlate due to differential mRNA turnover, different translational efficiencies, RNA interference down-regulating effects, etc. Gene expression determined by qPCR on cDNA rather estimates the transcriptional levels i.e. the promoter strength and state of activation.

Being neuronally expressed and targeting neurotransmitter releasing vesicles, *SYN1* can be regarded as a marker of neuronal development, density, and integration. The foetal porcine brain becomes convoluted between embryonic day 60 and 80 but also develops beyond that point in time. The significant alterations in porcine *SYN1* expression observed during the embryonic stages in the various brain compartments most likely reflect the different biological functions of these compartments. Hence, CBE, BST, and BSG serve more basal purposes in nerve signal transmission and e.g. mediate controlled movements, seem to stagnate earlier developmentally, and may also have a lower neuronal density. On the other hand FCO and HIP present a profile of increasing prenatal *SYN1* expression and likely also harbours relatively more neurons complying with the cognitive and memory functions of these tissues. The foetal porcine brain may thus provide an excellent model for studying prenatal brain and neuronal development due to its size, availability, and recapitulation of diseases of the human brain.

The porcine *SYN1* promoter was studied and evaluated for use in generating neurodegenerative disease models. From conservation of the nucleotide sequence in general and regulatory elements in particular, neuron-specificity can be anticipated also from the porcine *SYN1* promoter. The transgene expression level attainable with this promoter was estimated from quantification of endogenous *SYN1* mRNA found to be at the same order of magnitude as the GAPDH expression. Taking into account that (i) *SYN1* is expressed from only one allele, (ii) both *SYN1* transcripts are driven by the same promoter, and (iii) proven enhancer elements (i.e. the WPRE) can be an integral part of the transgene expression cassette, the porcine *SYN1* promoter presents as a good choice for establishing models of neurodegenerative diseases. The neuron-specificity of the human *SYN1* promoter has been demonstrated in both earlier and recent studies [18,51]. A very high specificity for neural expression was seen in rat neostriatum, thalamus and neocortex after lentiviral transfer of pSYN1:GFP constructs [18]. Similarly, in rat hippocampal/cortical embryonic neurons infected with lentivirus encoding pSYN1:GFP a neuron-specific expression was observed [51]. Neuron-specificity is presumably crucial for precise mimicking of disorders like Alzheimer's and Parkinson's disease by avoiding induction of pathological changes e.g. in glia as in multiple system atrophy or in any other somatic cells that could give rise to adverse phenotypes. Furthermore, a solid level of transgene expression even prenatally is desirable since over-expression of wild-type proteins is known solely to cause some degenerative disorders and since the diseases are by nature slowly progressing.

The presented *SYN1* promoter could be a good candidate for attempting to generate porcine models of human neurodegenerative disorders. As a model species of human disorders including those of neurodegenerative character, the pig is attracting attention for its anatomic, physiologic, and genetic homology to man.

We have analyzed the methylation status of the coding exons of the porcine *SYN1* gene and found significant difference between brain and liver with the highest degree of methylation in brain. A discrete sequence of the porcine *SYN1* promoter very close to the TSS was found to be completely unmethylated in occipital cortex and liver. Occipital cortex expresses high levels of *SYN1* transcript (Fig. 3) whereas no expression is found in liver. Similarly, no methylation was found in other promoter regions of *SYN1* in human and mouse [48]. A 104 bp DNA stretch in the *SYN1* promoter (see below) was also examined for methylation. Only two CpGs of 1,453 were found methylated in liver, yielding a methylation degree of 0%. Similarly, only two methylated CpGs out of 275 were identified in brain (occipital cortex). According to the classification [52] this means an unmethylated status (U:<20%). It is estimated that between 60% and 80% of all CpGs are methylated in mammals [53,54]. Unmethylated CpGs are often clustered in CpG islands (CGIs) in promoter regions of mainly house-keeping genes [55]. Saxonov et al. [56] estimated that approx. 70% of promoters belong to a class with high CpG content and around 30% are in a class with a CpG content characteristic of the overall genome i.e. a low CpG content. The linkage between gene promoter methylation and transcriptional suppression has been well recognized for several years. Generalized, genes with hypermethylated promoters are transcriptionally silent, and DNA methylation gradually accumulates upon long-term gene silencing [57]. In some cancers promoter CGIs become hypermethylated resulting in transcriptional silencing of tumor suppressor genes. From our data we conclude that the lack of expression of S*YN1* in liver is not the result of methylation of the promoter. DNA methylation represents a mechanism of epigenetic regulation in eukaryotes that is heritable thorough cell division. DNA methylation involves the addition of methyl groups to cytosine to form 5-methyl-cytosine and occurs almost exclusively within the context of CpG dinucleotides. It is estimated that 80% of all CpG sites in the human genome are methylated. CpG islands contain clusters of CpG dinucleotides which are often localized near the 5′ end of genes [58,59]. Methylation of CpG dinucleotides within promoter CpG islands is rare in normal tissue, but alterations in DNA methylation is frequent in diseases such as diabetes, schizophrenia, multiple sclerosis, and cancer [60,61]. Also, alterations in DNA methylation pattern is seen with increasing age in mice and human [62,63]. Our methylation study was hampered by the low number, one, of old pigs included. Therefore, the results can only be regarded as preliminary and hypothesis generating and studies of more animals are needed to certify the high methylation level in old pigs.

## Figures and Tables

**Table 1:** Methylation status of the porcine *SYN1* gene in liver and brain (Sus scrofa 10.2).

Gene	Length (bp)	Chr.	Start	End	Tissue	Methylated reads	Total reads	Methylation percentage
SYN1	51,915	X	47,336,723	47,388,638	Brain	5232	7948	66
					Liver	14,021	25,540	55
pSYN1^a^	113	X	47,336,576	47,336,689	Brain	2	275	0
					Liver	2	1453	0

a Methylation status of a discrete sequence of the of *SYN1* promoter in liver and brain (Sus scrofa 10.2).

## Appendix Supplementary materials

**Supplementary Fig. 1.** pSYN1-driven GFP expression in olfactory neurons. Ventral view of zebrafish embryo head, anterior to the top. The picture is representative of GFP positive Tg(pSYN1:GFP) embryos (*n* > 50) at 72 hpf. The embryo shows distinct GFP expression in olfactory neurons (ON) and retina (R) in addition to the signals shown in Fig. 5.

**Supplementary Fig. 2.** pSYN1:GFP expression co-localizes with neuronal marker. Z-projections of stacks from red (left panel) and green (mid panel) channels individually and merged (right panel). 40× magnification lateral image of the neural tube of an anti-GFP (green) anti-acetylated tubulin (red) immunostained zebrafish embryo 28 hpf. The images show a brightly GFP-labeled cell body with a weakly labeled axon co-localizing with acetylated tubulin.

**Table S1**. Exon/intron structure of the porcine *SYN1* gene.
