# Supplementary figures and images for: Porcine synapsin 1: SYN1 gene analysis and functional characterization of the promoter
Source: FEBS Open Bio. 2013 Oct 7;3:411–20. doi: 10.1016/j.fob.2013.10.002 (PMC3821028; doi:10.1016/j.fob.2013.10.002)

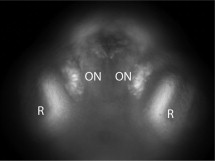

Supplement: Supplementary file 1 — Supplementary Fig. 1. pSYN1-driven GFP expression in olfactory neurons. Ventral view of zebrafish embryo head, anterior to the top. The picture is representative of GFP positive Tg(pSYN1:GFP) embryos (n > 50) at 72 hpf. The embryo shows distinct GFP expression in olfactory neurons (ON) and retina (R) in addition to the signals shown in Fig. 5. [file mmc1.jpg]

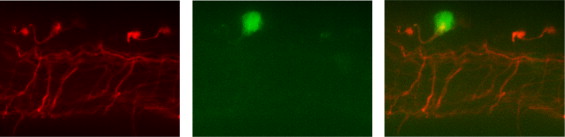

Supplement: Supplementary file 2 — Supplementary Fig. 2. pSYN1:GFP expression co-localizes with neuronal marker. Z-projections of stacks from red (left panel) and green (mid panel) channels individually and merged (right panel). 40× magnification lateral image of the neural tube of an anti-GFP (green) anti-acetylated tubulin (red) immunostained zebrafish embryo 28 hpf. The images show a brightly GFP-labeled cell body with a weakly labeled axon co-localizing with acetylated tubulin. [file mmc2.jpg]
