# Supplementary material for: Porcine synapsin 1: SYN1 gene analysis and functional characterization of the promoter
Source: FEBS Open Bio. 2013 Oct 7;3:411–20. doi: 10.1016/j.fob.2013.10.002 (PMC3821028; doi:10.1016/j.fob.2013.10.002)
Supplement: Supplementary file 3 — Table S1. Exon/intron structure of the porcine SYN1 gene. [file mmc3.pdf]

Table S1

Exon/intron structure of the porcine *SYN1* gene

	Porcine Size (bp)	5'-sequence	3'-sequence	Human Size bp)
Exon1	377*	ATGAACTAC	CACCGACTG	377*
Intron 1	12,782	GTAAGCCAT	CTTCTCTAG	12,153
Exon 2	58	GGCGAAATA	GTAGAACAG	58
Intron 2	103	GTTAGTTGG	CCTTCGCAG	104
Exon 3	92	GCTGAATTC	GGTCATGCG	92
Intron 3	2,171	GTGAGTATA	CCACCCCAG	1,565
Exon 4	157	GTCTCTGAA	CCCTGGGTG	157
Intron 4	171	GTAAGTGAC	CTCTCATAG	155
Exon 4	90	TTTGCCCAG	AAAGAGATG	90
Intron 5	27,764	GTGAGTCTT	CCCTACCAG	27,476
Exon 6	63	CTCAGCAGC	ATGGGCAAG	63
Intron 6	1,889	GTAAGACCC	CCCGCACAG	798
Exon 7	143	GTCAAGGTG	GGCCTACAT	143
Intron 7	91	GTAAGTGGA	GTTCTCCAG	91
Exon 8	75	GAGGACGTC	GTCTGACAG	75
Intron 8	98	GTGGGCGGC	TCTTGGCAG	98
Exon 9	103	GTACAAGCT	ATCATCGAG	103
Intron 9	1,562	GTGGGAGCC	CTCCCCCAG	856
Exon 10	147	GTGGTGGGC	CACAGCCAG	147
Intron 10	352	GTCAGGCCC	CTGCCGCAG	352
Exon 11	88	ACGCCGTCC	CACCGCAGG	88
Intron 11	106	GTAAGTGGG	GTCCCTCAG	97
Exon 12	610	GTGGCCCTC	CCAGCTCAA	589
Intron 12	1,662	GTAAGGGGA	TCTCTCTAG	1002
Exon 13	136**	CAAATCCCA	TCCGACTGA	136**

*5' coding sequence only
** 3' coding sequence only
